# Facile Fabrication of Methyl Gallate Encapsulated Folate ZIF-L Nanoframeworks as a pH Responsive Drug Delivery System for Anti-Biofilm and Anticancer Therapy

**DOI:** 10.3390/biomimetics7040242

**Published:** 2022-12-16

**Authors:** Saeed M. Marji, Mohammad F. Bayan, Abdolelah Jaradat

**Affiliations:** 1 Faculty of Pharmacy, Philadelphia University, P.O. Box 1, Amman 19392, Jordan; 2 Faculty of Pharmacy, Isra University, P.O. Box 33, Amman 11622, Jordan

**Keywords:** anticancer, antibiofilm, cytotoxicity, folic acid, pH responsive, zeolitic imidazole frameworks

## Abstract

Zeolitic imidazole frameworks are emerging materials and have been considered an efficient platform for biomedical applications. The present study highlights the simple fabrication of methyl gallate encapsulated folate-ZIF-L nanoframeworks (MG@Folate ZIF-L) by a simple synthesis. The nanoframeworks were characterized by different sophisticated instruments. In addition, the drug-releasing mechanism was evidenced by in vitro releasing kinetics at various pH conditions. The anti-biofilm potential confirmed by the biofilm architectural deformations against human infectious pathogens MRSA and N7 clinical strains. Furthermore, anticancer efficacy assessed against A549 lung cancer cells. The result reveals that the MG@Folate ZIF-L exposed a superior cytotoxic effect due to the pH-responsive and receptor-based drug-releasing mechanism. Based on the unique physicochemical and biological characteristics of nanoframeworks, it has overcome the problems of undesired side effects and uncontrolled drug release of existing drug delivery systems. Finally, the in vitro toxicity effect of MG@Folate ZIF-L was tested against the *Artemia salina* (*A. salina)* model organism, and the results show enhanced biocompatibility. Overall, the study suggested that the novel MG@Folate ZIF-L nanoframeworks is a suitable material for biomedical applications. It will be very helpful to the future design for targeted drug delivery systems.

## 1. Introduction

Zeolitic imidazole framework (ZIF) is an efficient material in biomedicine, due to its biodegradability, drug loading capacity, and stimuli-responsive drug release. It gives more advantages in the progressive development of stimuli-responsive drug delivery systems [1,2,3,4]. The remarkable physicochemical properties allow for the construction of the biomolecules’ encapsulated drug delivery system. The superior stability in physiological conditions and their acidic decomposition make them highly attractive cargo in pH-responsive drug delivery applications [5,6,7,8,9,10]. Recently, encapsulation of plant-derived compounds within ZIFs is an innovative process in human pathogenic disease and cancer treatments. The pH-responsive ZIFs are the best stimuli-based transporters in targeted drug delivery [11,12,13]. Modifying the surface of ZIFs with specific ligands activates the drug release mechanism when they reach the targeted cells. ZIFs release the targeted drug molecules in specific tumor environments. It is able to solve the drawbacks of existing drug delivery systems. In this context, surface modification of ZIFs with folic acid is a common strategy in cancer-targeted drug delivery. Naturally, folic acid acts as a receptor that can specifically adhere the targeted molecules to cancerous sites [12,13,14]. A folate receptor-mediated drug delivery system will promote drug internalization by folate receptor-mediated cancer cell endocytosis [15,16]. Recently, plant-derived compounds have received a lot of attention in cancer and antimicrobial applications. Methyl gallate is more efficient in antibacterial, antioxidant, antiviral, and anticancer activities [17,18,19,20,21,22,23]. Methyl gallate demonstrates severe cytotoxic effects in direct intravenous injection with poor water solubility constituting the disadvantages in medical usage [24,25,26]. Thus, this work focused on methyl gallate encapsulated folate ZIF-L nano frameworks synthesis and in vitro evaluations of the anti-biofilm and anticancer potential. This present research work mainly focused on the fabrication of drug delivery systems in biomedicine.

## 2. Materials and Methods

### 2.1. Chemicals Used

Zn(NO₃)₂, C_4_H_6_N_2_, Folic acid, Mueller Hinton agar, Nutrient agar, penicillin, streptomycin, Fetal bovine serum, DMEM high glucose medium, Crystal violet, Artemia cysts and Methyl gallate were purchased from Sigma Aldrich, Darmstadt, Germany. All the chemicals and reagents were used without purification.

### 2.2. Synthesis of MG@Folate ZIF-L Nanoframeworks

Methyl gallate encapsulated Folate ZIF-L nanoframeworks were prepared according to the previous report by Prabhu et al. [8] with some modifications. Briefly, 100 mg of zinc nitrate and 300 mg of methyl imidazole were dissolved in 50 mL of separate DD water. Furthermore, 20 mg of folic acid was dissolved in 10 mL of methanol concentration. Under the magnetic agitation, 2-methyl imidazole solution was slowly added to zinc nitrate solution, and the stirring was continued for another 30 min. Then, 10 mL of folic acid solution were added to zinc nitrate and the methyl imidazole mixture, and the magnetic stirring was continued for another hour. Its pale yellow coloured colloid formed indicates the formation of folate ZIF-L framework. After 60 min of magnetic agitation, 10 mL of methyl gallate solution was added drop by drop into the above folate ZIF-L colloid. Finally, the yellow-coloured colloid appeared. Centrifugation was used to eliminate the extra precursors by washing DD water through it. For six hours, the colloidal sample was dried at 60 °C. The alone Folate ZIF-L nanoframeworks were prepared using the above-mentioned method without the addition of methyl gallate.

### 2.3. Quantification of Drug Loading Capacity

A UV-Vis spectrometer was used to quantify the amount of methyl gallate that was loaded into the Folate ZIF-L nanocomposite. By using an HCl aqueous solution breakdown experiment, the total amount of methyl gallate loading in Folate ZIF-L was calculated. Utilizing the methyl gallate standard calibration curve, the quantification was completed. The drug loading capacity (DLC %) of fabricated material was calculated by the following equation:

(1) $$\mathrm{DLC}\left(\%\right)=\frac{\mathrm{The}\;\mathrm{amount}\;\mathrm{of}\;\mathrm{drug}\;\mathrm{loaded}}{\mathrm{Total}\;\mathrm{amount}}\times 100$$

A total drug loading (DLC) of 22.17% was determined for the MG@Folate ZIF-L nanoframework.

### 2.4. Physicochemical Characterization

The prepared samples were examined using a UV-Vis spectrophotometer to ascertain the characteristic absorption spectrum over a wavelength range of 200–800 nm. An X-ray powder diffractometer was used to determine the size and crystalline nature of the synthesized ZIF-L and FU@ZIF-L. (Ultima IV X-ray diffractometer, Rigaku, Japan). By using FTIR spectrum analysis with a frequency range of 4000–400 cm^−1^ and a resolution of 4 cm^−1^, the functional group of FU@ZIF-L was examined (IRAffinity-1 spectrophotometer, Japan′s Shimadzu, Kyoto, Japan). Scanning electron microscopy was used to evaluate the surface morphology of nanocomposite materials (Inspect F50, Eindhoven, The Netherlands). A transmission electron microscope was used to examine the size and crystal shape of FU@ZIF-L (FEI Morgani 268, Brielle, The Netherlands). Dynamic light scattering (DLS) analysis was used to determine the FU@ZIF-L nanocomposite′s particle size (Malvern zeta sizer, Malvern Panalytical Ltd., Malvern, UK).

### 2.5. Drug Release Study

The pH-responsive drug releasing kinetics of MG@Folate ZIF-L nanoframeworks were evaluated at different pH values (5, 6, and 7.4). The total amount of drug-releasing at every pH was calculated from the calibration graph of methyl gallate organized by maximum absorbance at 270 nm in the treated solutions. A sample of (50 mg) was dissolved in PBS buffer solution (100 mL) and subjected to magnetic agitation at 250 rpm speed at 37 °C. The test solution was taken from the mother solutions of MG@Folate ZIF-L suspended PBS buffer solution.

### 2.6. Artemia salina Acute Toxicity Bioassay

Using *Artemia salina* as a model system, the lethality bioassay was used to evaluate the cellular toxic effect of MG@Folate ZIF-L [27]. Freshly hatched *A. salina* nauplii were separated into six groups each containing (*n* = 20). They were placed in 12 well plates and subjected to *A. salina* and treated with different concentrations (25–150 µg/mL) of MG@Folate ZIF-L in seawater for 24 h. The well-containing *A. salina* in seawater without nanoframeworks is considered a negative control. The number of surviving and dead organisms was counted by using a normal magnification lens. By comparing the number of deaths and survivors in the control and control groups, the LC50 value for treated *A. salina* nauplii was determined using Abbott′s formula:

$$\%\;\mathrm{of}\;\mathrm{lethality}=\frac{\mathrm{Nt}-\mathrm{Nc}}{\mathrm{Nc}}\times 100$$

Nt and Nc—Number of alive *A. salina* naupli in treated and control groups.

### 2.7. Anti-Biofilm Activity of MG@Folate ZIF-L Nanoframeworks

Anti-biofilm proficiency of MG@Folate ZIF-L was investigated against infectious bacterial strains (MRSA and N7) and was evaluated according to the method of Prabhu et al. [11]. After the overnight culture of MRSA and *E. coli*, bacterial strains were subjected to dilution (108 CFU/mL) in TSB broth (0.5% glucose) followed by treatment with different doses of MG@Folate ZIF-L (20–100 µg/mL) for 48 h at 37 °C. PBS was used to get rid of the floating bacteria. A 0.4% crystal violet solution was used to stain the microscopic slides on which the biofilm had developed. Washing three times with DD water and 10% glacial acetic acid for 10 min removed extra stains. The bio-film inhibition was measured by quantification method from the color intensity of the treated bacterial medium at an absorbance of 570 nm using multiple readers in UV-Vis spectroscopy.

### 2.8. Microscopic Observation of Bio-Film Morphology

Architectural and morphological alteration of MG@Folate ZIF-L treated bacteria was examined, and images were recorded by inverted phase contrast microscopy. Treated bio-film glass slides were subjected to 0.2% of crystal violet (CV). The excess amount of CV stain was detached by washing, and bio-film matrix damage is identified under an inverted light microscope (Lawerence & Mayo, Mumbai, India) at 40× magnification.

### 2.9. Assessment of Cytotoxic Effect against Lung Cancer Cells

The cytotoxic effect of MG@Folate ZIF-L against A549 lung cancer cells was evaluated by using an MTT assay according to Prabhu et al. [8]. The A549 cells were allowed to be growing overnight culture. After the overnight culture, cells were treated with various concentrations (10 to 100 µg/mL) of MG@Folate ZIF-L at 37 °C for 24 h. Following the administration of MG@Folate, ZIF-L were treated with MTT (1 mM) for 4 h at 37 °C. Formazan was then dissolved, producing a blue color in DMSO, and the OD value was measured at 570 nm using a multi-plate reader (Molecular Device Spectramax M3, Molecular Devices, LLC., 3860 N First Street, San Jose, CA 95134 USA). The percentage of cell viable cells was calculated from the triplicate values.

### 2.10. Assessment of ROS Generation by MG@Folate ZIF-L Treated Cells

The mitochondrial reactive oxygen species generation (ROS) of MG@Folate ZIF-L nanoframeworks treated cells was determined by DCFH-DA (2,7-diacetyl dichloro fluorescein acetate) fluorescence dye assay [28]. Pre-seeded cells were treated with MG@Folate ZIF-L nanoframeworks at its IC50 concentration (IC50 34.5 ± 2.14 µg/mL) for 24 h followed by treatment with DCFH-DA (50 µL) at 37 °C for 30 min. The fluorescence intensity was quantified by Cary Eclipse, a fluorescence spectrophotometer with a wavelength of 480/530 nm (emission/excitation nm). Fluorescence images of treated cells were taken under a fluorescent microscope (Carl Zeiss Axio Observer Z1, Jena, Germany) at a magnification 20×.

### 2.11. Assessment of Nuclear Damage Using Hoechst Staining

Hoechst staining assay was used to evaluate the genotoxicity of A549 lung cancer cells generated by MG@Folate ZIF-L [8]. Hoechst 33258 staining (2 mg/mL) was applied to adhered cells that had been treated with MG@Folate ZIF-L at its IC50 concentration for 20 min. A spectrofluorimeter was used to detect the intensity of fluorescence at an emission/excitation wavelength of 350/460 nm after an excessive amount of stain was washed away using PBS solution. Fluorescent microscopy at 20× was used to take pictures of individual cells.

### 2.12. Statistical Analysis

Probit analysis software was used to compare the control and treatment groups and calculate the IC 50, LD50, and LD90 values. (one-way analysis of variance (ANOVA) using SPSS 17.0 software). Significance was at the level of *p* < 0.05 and mean ± S.D. calculated from triplicate values.

## 3. Results and Discussion

### 3.1. UV-Vis Spectroscopy Analysis

**Figure 1:**
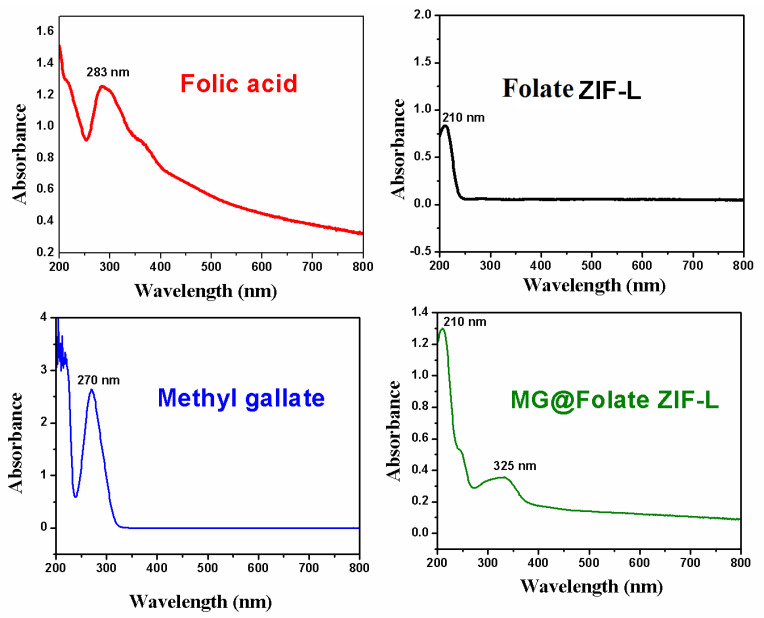
UV-Vis spectrum of folic acid (red) Folate ZIF-L (black), MG@Folate ZIF-L (green), methyl gallate (blue).

The characteristic absorption spectra of bare ZIF-L and folic acid showed a peak at 210 and 283 nm, respectively (Figure 1). Alone, methyl gallate shows the absorbance at 270 nm. Methyl gallate encapsulated Folate ZIF-L showed the absorption peak at both 210 and 325 nm, respectively. The shift in the absorption spectra of MG@Folate ZIF-L nanoframeworks confirmed the encapsulation of methyl gallate in the Folate ZIF-L nano framework. Band gap energy of Folate ZIF-L and MG@Folate ZIF-L was calculated to be 5.41 eV and 5.75 eV, respectively. The encapsulation of organic substances such as methyl gallate and folic acid, which decreases the particle size of nano frameworks, improved the band gap energy.

### 3.2. Powder XRD Analysis

**Figure 2:**
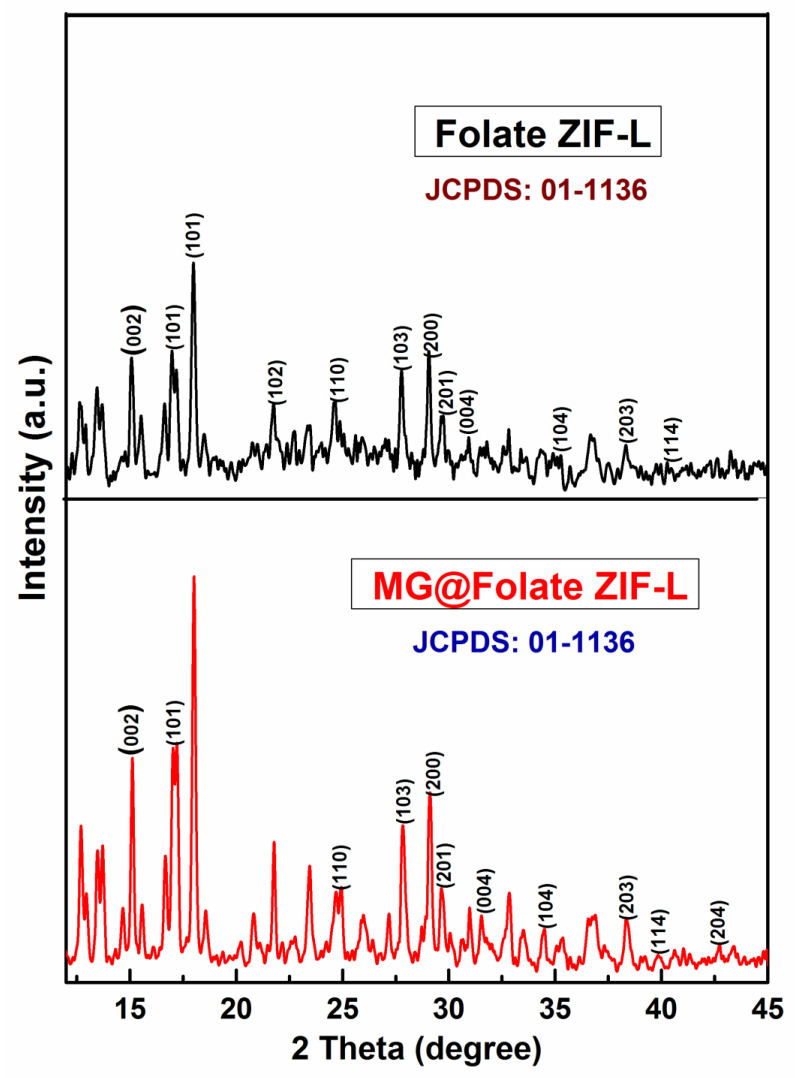
Powder XRD analysis of Folate ZIF-L (black) and MG@Folate ZIF-L (red).

The powder XRD spectra of MG@Folate ZIF-L nanoframeworks reveals the crystalline nature of the prepared material. Sharp diffraction peaks at around 15.04°, 16.86°, 21.86°, 24.65°, 27.84°, 29.17°, 30.72°, 32.45°, 35.33°, 36.71°, 40.97°, 43.30° and 46.13° corresponding to (002), (101), (102), (110), (103), (200), (201), (004), (104), (203), (114), (204) and (213) Braggs reflection planes (Figure 2). According to JCPDS No. 01-1136, diffraction peaks at 2θ values and planes represent the two-dimensional flake-like crystal structure. Scherer′s formula has been used to calculate the crystalline size of MG@Folate ZIF-L nanoframeworks, which is approximately 48.26 nm.

### 3.3. FTIR Analysis

**Figure 3:**
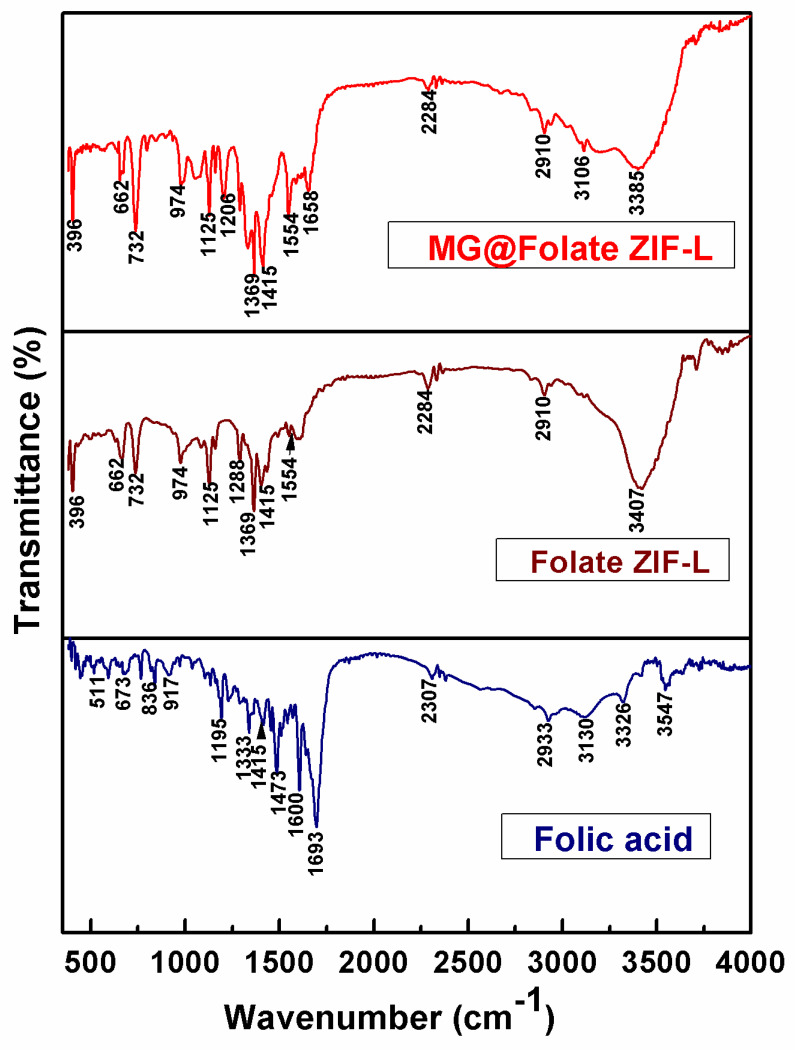
FTIR spectrum of Folate ZIF-L (brown), MG@Folate ZIF-L (red) and folic acid (blue).

The inclusion of folic acid and methyl gallate active groups in bare Folate ZIF-L and MG@Folate ZIF-L was confirmed by FTIR spectroscopy. The FTIR spectra of MG@Folate ZIF-L and Folate ZIF-L are shown in Figure 3. With a few additional peaks that show the loading of methyl gallate, both are remarkably comparable. The Zn-N stretching vibrations’ peaks at 396 and 662 cm^−1^ correlate to the synthesis of ZIF-L between zinc and imid-azole. The C-N stretching vibration of imidazole linkers in Folate ZIF-L and MG@Folate ZIF-L nanoframeworks was confirmed by intense peaks at 974 cm^−1^, 1125 cm^−1^, and 1154 cm^−1^. Stretching vibrations of the C=O molecule correspond to the spectral bands at 1554 and 1658 cm^−1^. The N-H group of the imidazole linker is shown by a tiny peak at about 2910 and 2933 cm^−1^. The O-H bending vibration of the Folate ZIF-L and MG@Folate ZIF-L nanoframeworks is responsible for peaks at about 3385 and 3547 cm^−1^.

### 3.4. Chemical Composition and Morphological Analysis

**Figure 4:**
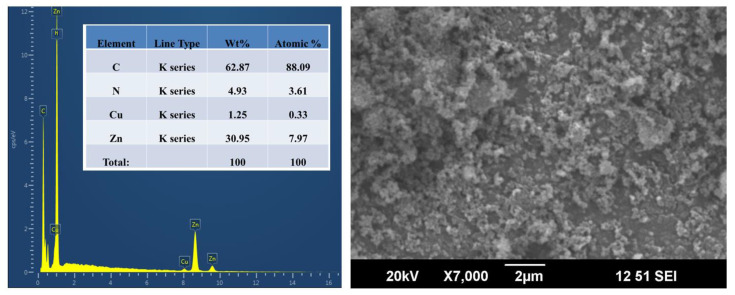
SEM and EDX spectrum of MG@Folate ZIF-L nanoframeworks.

The elemental constituents and surface morphology of MG@Folate ZIF-L were examined by using EDX techniques and SEM analysis. The microscopic images of MG@Folate ZIF-L reveal that agglomerated spongy crystal-like structure (Figure 4). In the simultaneous one-pot synthesis method, the addition of folic acid and methyl gallate to ZIF-L frameworks interrupts the crystal uniformity of ZIF-L, thereby reducing the crystal size. EDX spectral analysis of MG@Folate ZIF-L revealed that no other metal element was present in the prepared nano frameworks. The total weight percentage of zinc in MG@Folate ZIF-L is 30.95%, respectively. A higher amount of carbon 62% confirmed the richness of organic compounds availability in nanoframeworks. The notable weight percentage of nitrogen 4.93% represents the coordination of zinc ions with the nitrogen of 2-methyl imidazole linkers.

### 3.5. TEM and DLS Analysis

**Figure 5:**
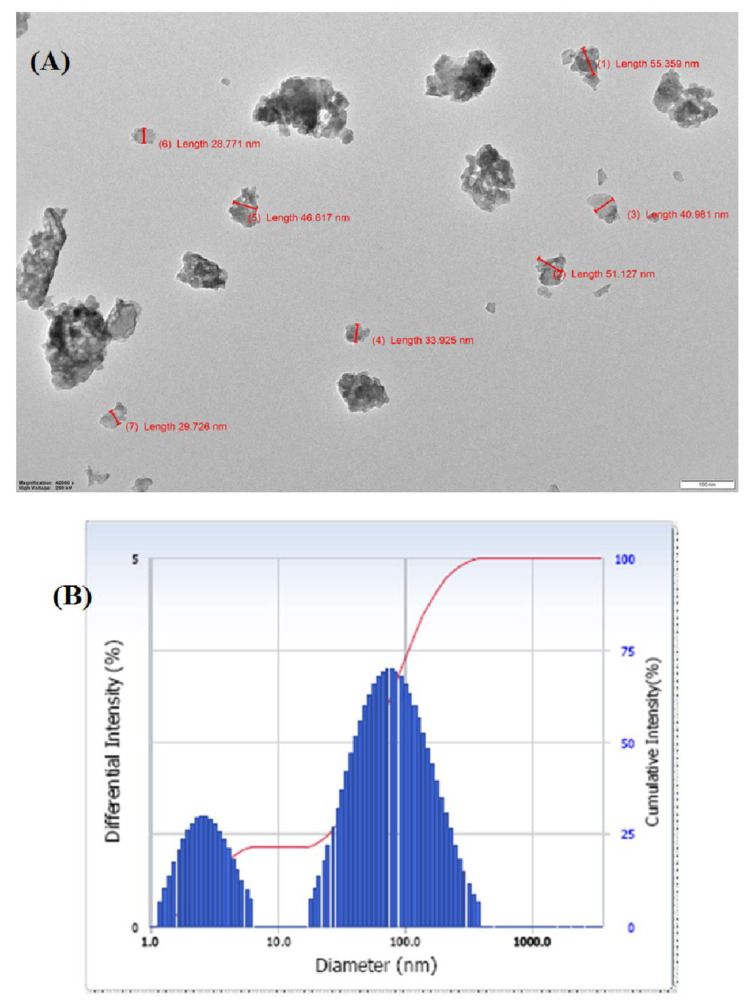
(**A**) TEM image of MG@Folate ZIF-L and (**B**) particle size analysis of MG@Folate ZIF-L by DLS technique.

Figure 5 displays the HR-TEM pictures of the synthesized MG@Folate ZIF-L micro frameworks (A). Agglomerated flakes were visible in the TEM picture of the MG@Folate ZIF-L. It was planned for MG@Folate ZIF-L to have an average particle size of 41.8 nm. The average diameter of the synthetic material in solution was shown in Figure 5B. The average size, which was found to be 75.2 nm, is suitable for allowing nanoframeworks to enter cells through endocytosis or adsorption. MG@Folate ZIF-L frameworks are an excellent candidate for drug delivery because of their nano size.

### 3.6. pH-Responsive Drug Releasing Mechanism of MG@Folate ZIF-L Nanocomposite

**Figure 6:**
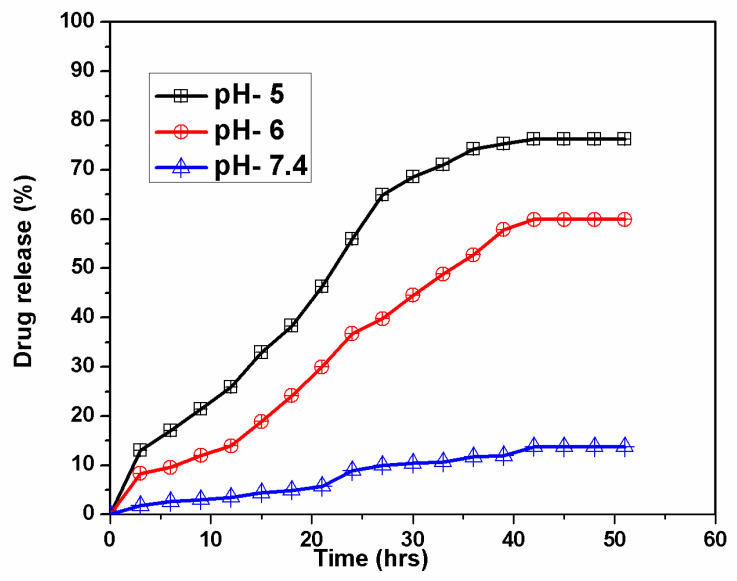
Cumulative drug release of methyl gallate at different pH (5, 6 and 7.4).

Stimuli-based drug delivery techniques overcome the drawbacks of classical methods, by efficient drug release on specific cancer sites, hence pH-responsive is a trusted and simple technique for cancer drug delivery application. In zeolitic imidazole frameworks, the organic linker imidazole and zinc iron covalent bonds get cleaved in acidic conditions [5,8,29]. Figure 6 shows the gradual increases in methyl gallate at acidic pH 5 and 6, which reached the stationary phase within the 39 h without changes up to 52 h. However, at the normal physiological pH of 7.4, a very minimum drug release was observed within the transit time. At neutral pH of 7.4, the MG@Folate ZIF-L showed a stable and uniform drug release capacity of 13.71%. When the pH of the medium was reduced to acidic conditions 5 and 6, the drug release capacity was gradually raised to 76.32 and 59.81% in the short period of 39 h (Figure 6). The enhanced drug release at lower pH indicates the cleavage of zinc and imidazole bonding from ZIF-L frameworks. Present results revealed that an MG@Folate ZIF-L nanoframework is a suitable drug delivery system for pH-responsive anticancer treatments.

### 3.7. Cytotoxicity of Folate ZIF-L Nanocomposite on Artemia salina

**Figure 7:**
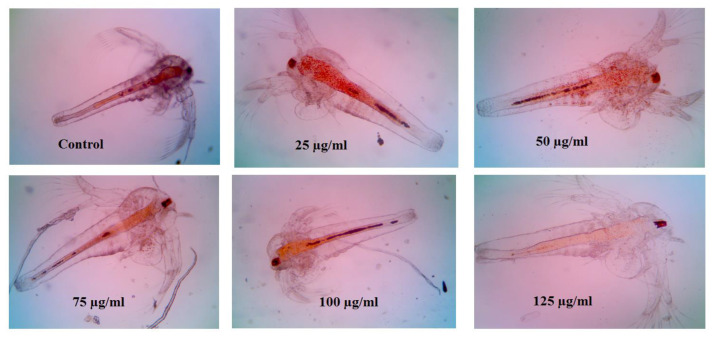
Cytotoxicity evaluation of *A. salina* nauplii post exposure to MG@Folate ZIF-L nanoframeworks (magnification 10×).

*A. salina nauplii* lethality assay is one of the reliable alternatives for MTT and other in vivo models. In this present evaluation, synthesized nanoframeworks revealed that the toxicity of MG@Folate ZIF-L nanoframeworks is more biocompatible. The cytotoxicity of MG@Folate ZIF-L nanoframeworks was quantified from death and survival of treated nauplii (Figure 7). The LC50 was calculated to be 118.43 ± 2.52 µg/mL. The growth inhibition and morphological changes of *A. nauplii* were identified under the inverted microscope, which shows much less toxicity and no significant changes in morphology and growth up to 125 µg/mL some deaths appeared. Our toxicity study revealed that MG@Folate ZIF-L nanoframeworks exhibited non-toxic behavior against *A. salina* at lower concentrations, which shows mild toxicity in higher doses (125 µg/mL). There is no significant modification in the swimming behavior of treated nauplii at the end of 48 h of testing duration.

### 3.8. Anti-Biofilm Activity of MG@Folate ZIF-L Nanoframeworks

**Figure 8:**
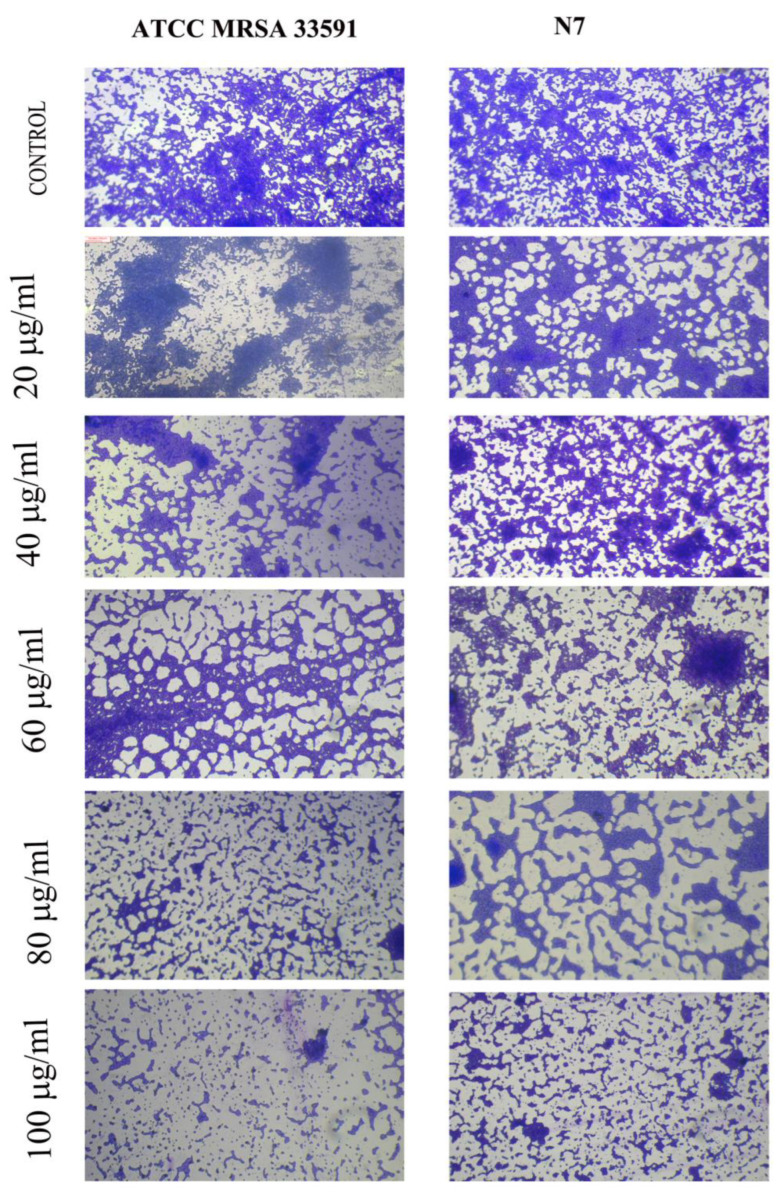
Light microscopic images anti-biofilm formed bacteria after treatment of MG@Folat ZIF-L nanoframeworks in different concentrations.

**Figure 9:**
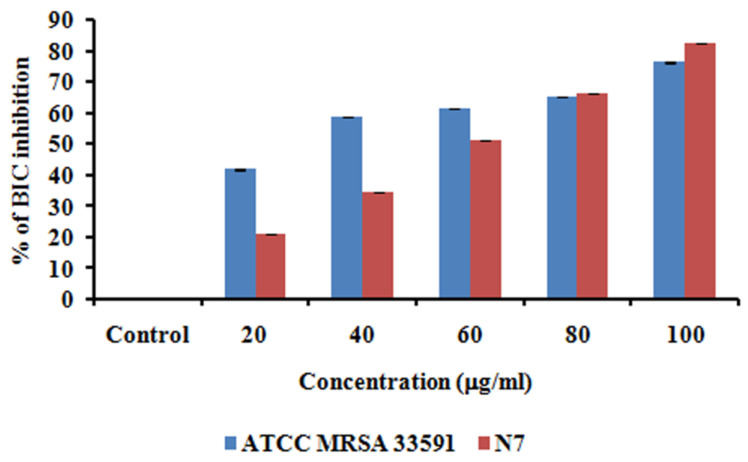
Quantification of biofilm inhibition potential of MG@Folate ZIF-L by crystal violet assay.

*Staphylococcus aureus* is a more dreadful clinical pathogen affecting humans, causing cruel infections and life-threatening diseases. The biofilm forming potential of MRSA provides antibiotic resistance to bacterial cells [30,31,32]. It is associated with unhygienic medical procedures like the use of impure surgical devices and touching people in the crowd. Advancement in the nanomedicine field has diverted the attention of nanotechnology researchers toward the annihilation of bio-film-causing bacteria via nano formulation [33,34]. The anti-biofilm potential of methyl gallate encapsulated Folate ZIF-L was assessed by the measuring color intensity of crystal violet present on the treated cells of MRSA (control—clinical strain—N7, ATC C MRSA 33591). Figure 8 illustrated that MG@Folate ZIF-L nanoframeworks prevent and eradicate MRSA 33591 and clinical strain N7 bio-film formation depending on the doses. Figure 9 showed the quantification of biofilm inhibition by the nanoframeworks at different concentrations, which concluded that MG@Folate ZIF-L efficiently controls the bacterial growth rate. Methyl gallate encapsulation within Folate ZIF-L enhanced the anti-biofilm efficacy of Folate ZIF-L by the adhesion of MRSA and N7 biofilm surfaces, thereby eradicating bio-film formation.

### 3.9. Microscopic Visualization of MG@Folate ZIF-L Nanoframeworks Treated MRSA and N7 Bio-Films

The microscopic observation of nanoframeworks treated bacterial slides (Figure 8) shows the depletion of bio-film growth and also architectural deformation on biofilm surfaces. However, the control slides showed dense biofilm and uniform architecture in both MRSA and N7 strains. The results reveal that the methyl gallate-loaded Folate ZIF-L nanoframeworks efficiently control the bio-film growth and break the cell walls of the bacterial colonies in dose depending on mode.

### 3.10. Anticancer Potential of MG@Folate ZIF-L Nanoframeworks

Lung cancer is a death-dealing form of human disease worldwide. Current therapeutic techniques such as chemotherapy, radiation, and surgery have increased the risk to patient health. Due to these classical treatment strategies, the patient immune system is damaged by the drugs, which leads to side effects. Recently, biomedical researchers have had a great interest in the uses of targeted drug delivery methods in cancer treatments [35,36,37,38]. Today, several synthetic drugs are available in the market for lung cancer cells targeting epidermal growth factor receptor (EGFR). These EGFR inhibits cancer cell growth and delays the spreading of infected cells. In some lung cancer cases, cancer cells developed resistance to epidermal growth factor receptor (EGFR), and these problems promoted a need for new drug development [39,40,41,42].

**Figure 10:**
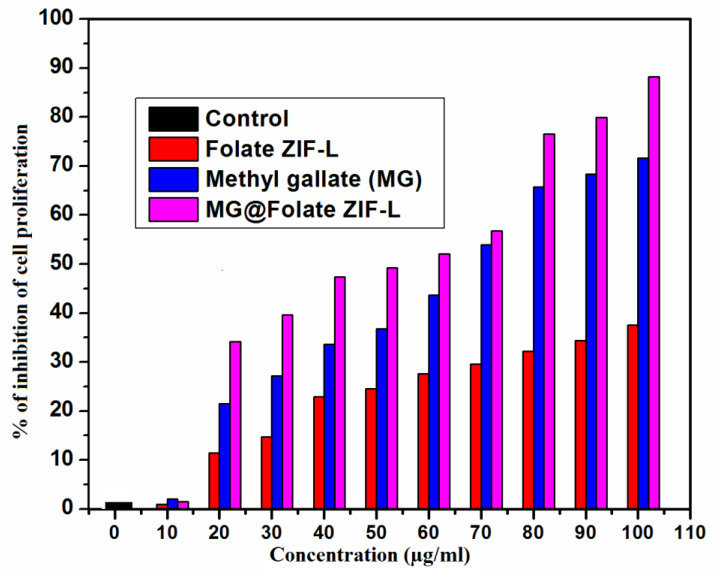
Assessment of cytotoxic effect of MG@Folate ZIF-L nanoframeworks against A549 lung cancer cells by MTT assay.

**Figure 11:**
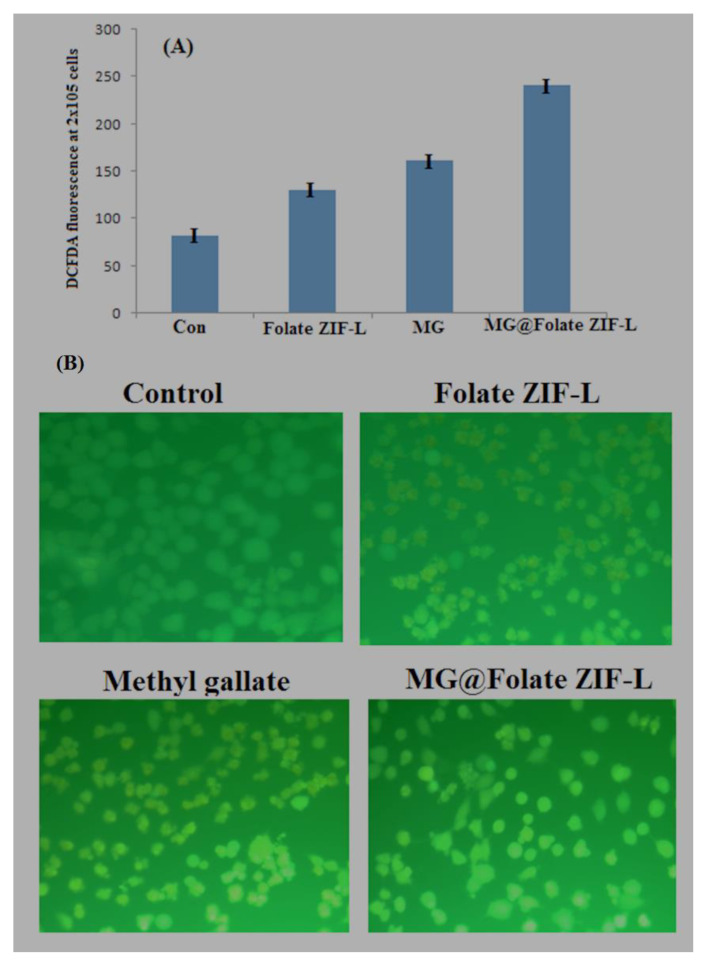
(**A**) Quantification of ROS level in A549 cells using fluorescence spectroscopy; (**B**) microscopic images of A549 cells post exposure of MG@Folate ZIF-L nanoframeworks induced intracellular ROS.

In this study, ZIF-L as a folic acid receptor bonded carrier for methyl gallate molecule delivery depends on the pH-responsive condition. By using the MTT assay on A549 lung cancer cells, the anti-proliferative effectiveness of free methyl gallate, Folate ZIF-L, and MG@Folate ZIF-L was determined. At the maximum dose (100 mg/mL), the results showed that Folate ZIF-L had a minimal cytotoxic impact. In this context alone, methyl gallate and MG@Folate ZIF-L show reliable inhibition at the same concentration, which increases cytotoxicity with an IC 50 value of 62.32 ± 0.08 mg/mL, respectively (Figure 10). Folate ZIF-L nanocomposite exhibits higher cytotoxicity at the highest dose of 100 mg/mL, when compared to the same dose of methyl gallate encapsulated Folate ZIF-L nanoframeworks, revealing the fact that Folate ZIF-L retains the lethality effect of methyl gallate incorporation. Structural changes in the A549 cells treated with MG@Folate ZIF-L nanoframeworks were identified by inverted phase contrast microscopy. Results concluded that (Figure 11B) both methyl gallate and MG@Folate ZIF-L treated cells exhibit membrane damages and cell shrinking with condensed chromatin, while the control cells exhibited normal cell morphology, and Folate ZIF-L treated cells show mild changes in cell morphology. The MG@Folate ZIF-L treated cells showed effective depletion in cell counting compared to the control group.

Nuclear morphological changes of MG@Folate ZIF-l treated and control cells were assessed from the Hoechst 33342 staining assay. Control cells show regular cell structure and spherical nuclei. However, Folate ZIF-L and methyl gallate treated cells showed mild alteration in the nuclei region. Figure 11B exposed high blue fluorescence, which indicates the fragmented nuclei. At the same time, MG@Folate ZIF-L induces condense nuclei leading the apoptosis. The percentage of normal and abnormal nuclei was evaluated by the quantification method. Figure 11A shows MG@Folate ZIF-L and methyl gallate treated cells showed 83.10 ± 1.32% of abnormal and 17.013 ± 1.31% of normal nuclei cells; when compared to control and Folate ZIF-L, the nuclear damage rate is higher in MG@Folate ZIF-L treated cells. Overall, the results confirmed that the MG@Folate ZIF-L treated cells show a high level of ROS generation, provoking oxidative stress-mediated genotoxicity and mitochondrial dysfunction ultimately leading the apoptosis process.

**Figure 12:**
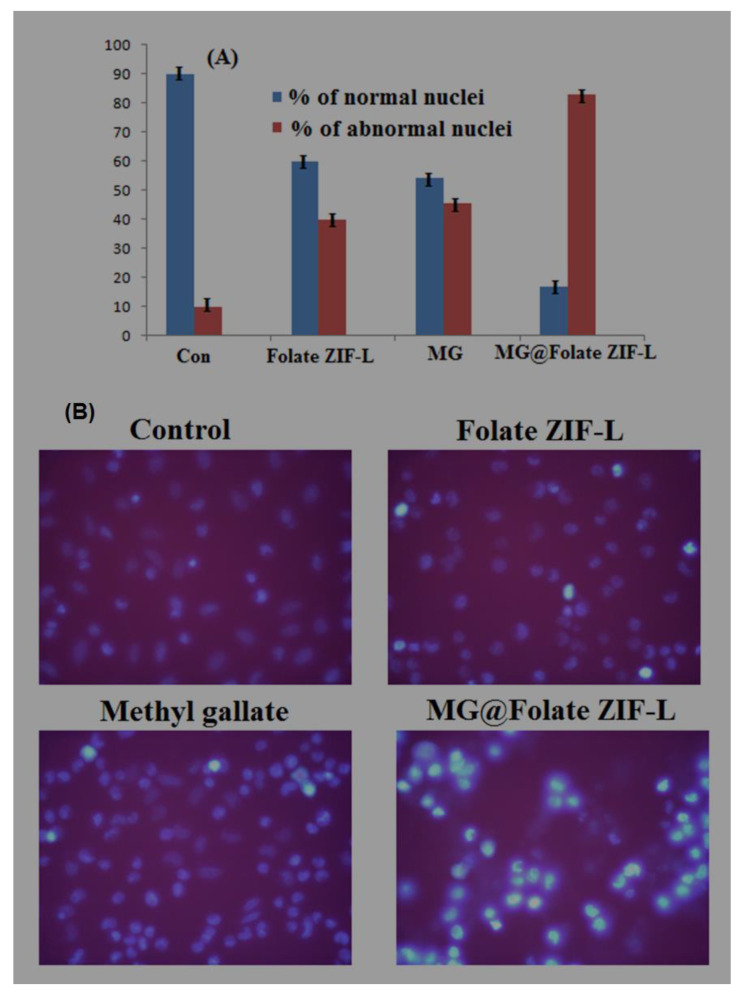
(**A**) Quantitative evaluation of normal and abnormal nuclei; (**B**) microscopic images of nuclear morphology changes of cells treated with MG@Folate ZIF-L nanoframeworks.

Anticancer efficacy of Chemotherapeutics depends on the generation of reactive oxygen species ROS mediated cell death mechanism. However, a variety of new drugs designing and treatment methods are based on targeting intracellular ROS levels of cancer cells [43,44]. Based on this concept, the generation of intracellular ROS level of lung cancer cells was assessed by DCFDH-DA fluorescence assay. Results revealed that the MG@Folate ZIF-L treated cells generate more cells DCF fluorescence intensity compared to Folate ZIF-L and vehicle control (Figure 12B). Spectrofluorimetric results concluded that the MG@Folate ZIF-L treated cells had five-fold increases in fluorescence intensity compared with control cells and a threefold increase compared to methyl gallate alone (Figure 12A). The releasing of methyl gallate and folic acid molecules leads cell wall damage and increases the intracellular ROS level.

## 4. Conclusions

The present study revealed the simultaneous encapsulation of folic acid and methyl gallate within the ZIF-L frameworks. The simple room temperature synthesis of MG@Folate ZIF-L is a reliable and eco-friendly approach compared to other existing classical methods. The encapsulation of folic acid and methyl gallate within ZIF-L frameworks enhanced the anticancer activity in human lung cancer cells based on the pH-responsive drug release mechanism. The MG@Folate ZIF-L nanoframeworks exhibited excellent anti-biofilm activity against human pathogens MRSA and N7 bacterial strains. The MG@Folate ZIF-L acts as an effective and biocompatible drug delivery system for the control of cancer cell proliferation and bio-film growth. After the pre-clinical trials, it might be an efficient and low-cost material for lung cancer therapy.

## Data Availability

All data are available in the manuscript.
